# Clinical value of auditory nerve enhancement in idiopathic sudden sensorineural hearing loss: a retrospective study

**DOI:** 10.3389/fneur.2024.1410516

**Published:** 2024-05-31

**Authors:** Xiao Sun, Lei Chen, Na Hu, Wenping Xiong, Yingjun Wang, Kunpeng Lu, Fujia Duan, Haibo Wang, Mingming Wang

**Affiliations:** ^1^Department of Otology Medicine, Shandong Provincial ENT Hospital, Shandong University, Jinan, China; ^2^Department of Otolaryngology Head and Neck Surgery, Shandong Provincial ENT Hospital, Shandong University, Jinan, China; ^3^Department of Radiology, Shandong Provincial ENT Hospital, Shandong University, Jinan, China

**Keywords:** MRI, hearing loss, sudden, auditory nerve enhancement, clinical features, prognosis

## Abstract

**Background:**

The pathogenesis of idiopathic sudden sensorineural hearing loss remains unclear, and no substantial breakthroughs have been achieved in its treatment. Therefore, we conducted this study with the aim to investigate the clinical features and prognostic factors of patients with idiopathic sudden sensorineural hearing loss and auditory nerve enhancement by using three-dimensional fluid-attenuated inversion recovery (3D-FLAIR) magnetic resonance imaging (MRI) of the inner ear.

**Methods:**

We retrospectively analyzed the clinical data of adult patients, who experienced sudden unilateral deafness and were admitted to the Department of Otolaryngology, Shandong Provincial ENT Hospital, between December 2020 and July 2021. Patients were divided into an auditory nerve enhancement group and a normal inner ear group, according to 3D-FLAIR MRI findings. Differences in sex, age, side, disease course, underlying diseases, dizziness/vertigo, vestibular function, degree of deafness, hearing classification, and treatment efficacy were analyzed.

**Results:**

Of the 112 cases of sudden idiopathic deafness, 16.07% exhibited enhancement of the auditory nerve on inner-ear 3D-FLAIR MRI. Statistically significant differences in the degree and type of hearing loss were detected between the two groups (*p* < 0.05). The rates of abnormal results in the caloric, vestibular-evoked myogenic potential, and video head impulse tests were higher in the auditory nerve enhancement group. The cure rate (11.1%) in patients with auditory nerve enhancement was lower than that in patients with normal inner ear MRI findings (28.7%); however, the difference was not statistically significant.

**Conclusion:**

Findings from 3D-FLAIR MRI scans of the inner ear indicated that patients with sudden deafness and auditory nerve enhancement experienced severe hearing loss, aggravated vestibular function injury, and a significantly decreased cure rate. Prompt treatment, ideally within 2 weeks of disease onset, can facilitate hearing recovery.

## 1 Introduction

Idiopathic sudden sensorineural hearing loss (ISSNHL) refers to sudden and unexplained sensorineural hearing loss within 72 h, experiencing a hearing loss of ≥30 dB HL at a minimum of three contiguous frequencies (1). With the rapid pace of life, the annual incidence of ISSNHL has increased in recent years, and patients tend to be younger. However, the pathogenesis of this sudden hearing loss remains unclear. The special anatomical position of the inner ear and limited drug delivery routes pose challenges for the diagnosis and treatment of ISSNHL. No significant breakthroughs have been achieved in the treatment of ISSNHL. Therefore, the pathological mechanisms of ISSNHL must be clarified for its successful treatment.

Recent studies have shown that modern magnetic resonance imaging (MRI) can be used to detect abnormal changes in the labyrinth of the inner ear. An increasing number of studies have demonstrated that 3.0 T MRI, particularly, the three-dimensional fluid-attenuated inversion recovery (3D-FLAIR) MRI detects inner signal changes in ISSNHL with satisfactory sensitivity and specificity (2, 3). The 3D-FLAIR MRI findings have been shown to correlate with the clinical features and prognosis of ISSNHL (2–6). The application of 3D-FLAIR MRI to detect different pathological changes in the inner ear, inner ear bleeding, and abnormal protein deposition may serve as a new objective indicator of ISSNHL prognosis (6, 7). With the advancement of various imaging technologies, 3D-FLAIR MRI after gadolinium contrast enhancement can be employed to assess inner ear blood loss and the permeability of the blood–nerve barrier (4–8). It can also be used to monitor pathological modifications in the auditory nerve, thereby evaluating the pathological mechanisms and prognosis of ISSNHL, which has a potential clinical value.

In this study, we aimed to investigate the clinical features and prognostic factors of patients with ISSNHL using 3D-FLAIR MRI after gadolinium enhancement of the inner ear auditory nerve.

## 2 Materials and methods

### 2.1 Case data

We retrospectively analyzed adult patients with unilateral ISSNHL who were admitted to the Department of Otolaryngology of Shandong Province between December 2020 and July 2021. The inclusion criteria were as follows: (1) meeting the diagnostic criteria for ISSNHL: sudden, unexplained sensorineural hearing loss within 72 h, with a hearing loss of ≥30 dB HL in at least three contiguous frequencies; and (2) having unilateral ISSNHL, first onset, with the course of disease ≤1 month. The exclusion criteria were as follows: (1) middle ear disease, retro-cochlear disease, Meniere’s disease, large vestibular aqueduct syndrome, genetic factors, and other pathogenic factors; (2) no treatment before admission; (3) pregnancy; or (4) contraindications for corticosteroid use.

### 2.2 MRI examination and grouping

**Figure 1:**
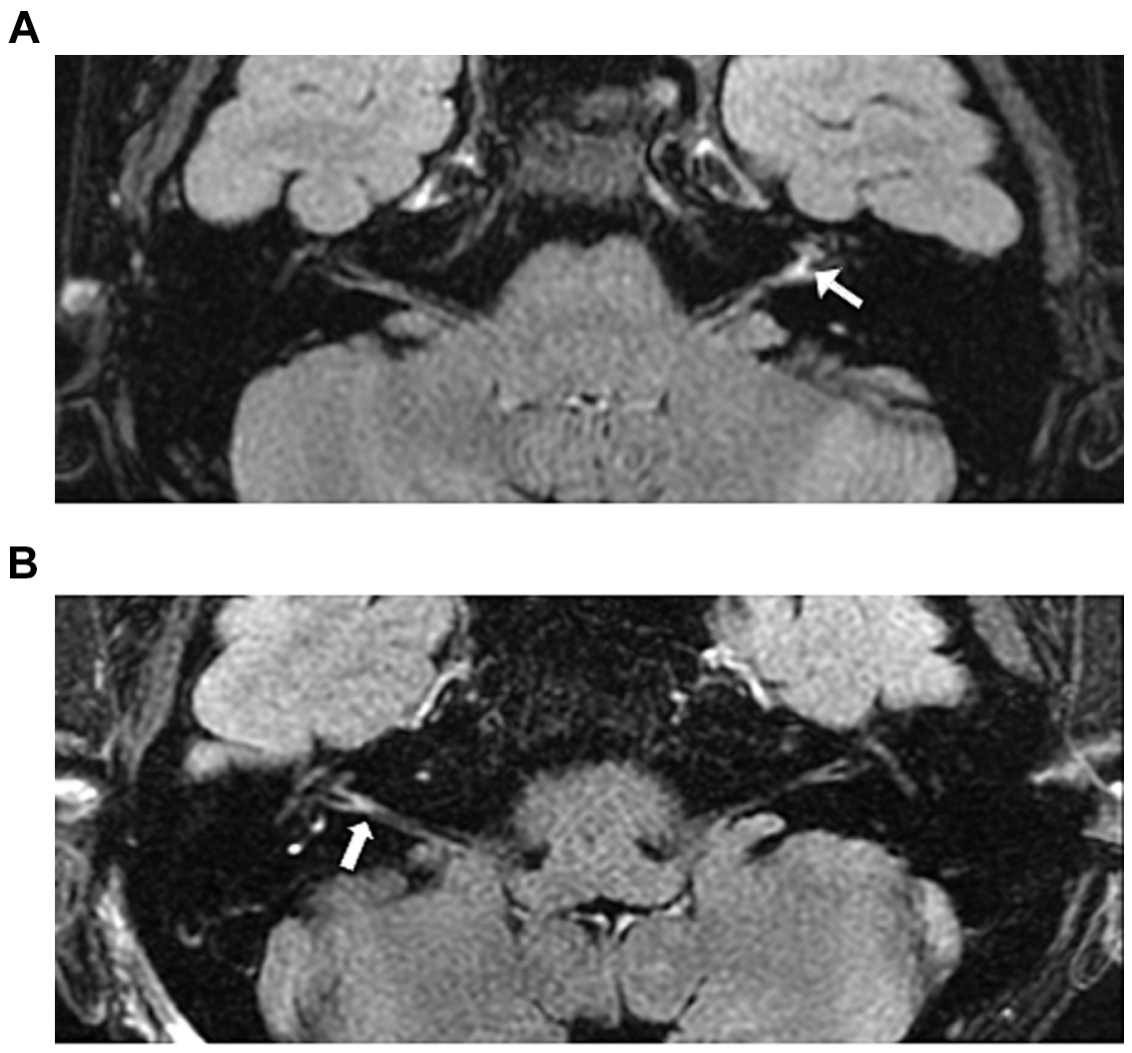
Examples of patients with profound hearing loss. **(A)** A 20-year-old woman with profound hearing loss in the left ear. The 3D-FLAIR (15 min + C) showed abnormally high signals within the cochlea, cochlear nerve, and vestibular nerve. **(B)** A 37-year-old woman presented with profound hearing loss in the right ear. The 3D-FLAIR (15 min + C) shows abnormally high signals in the cochlea, vestibular, semicircular canal, cochlear nerve, and vestibular nerve.

All the patients underwent plain and enhanced 3D-FLAIR MRI of the inner ear after admission. Internal auditory tract scanning was conducted using an 8-channel phased array coil and a 3.0 T magnetic resonance to acquire fat-suppressed fast spin-echo 3D-FLAIR T2-weighted, as well as fast spin-echo 3D T1-weighted and T2-weighted images. The contrast agent (0.2 mL/kg body weight gadolinium) was administered intravenously (the enhanced scan image is indicated by “C”), and the scan was performed after 15 min, 1 h, and 4 h (15 min yielded the best results). Two senior imaging technicians issued a report for each patient, and the patients were categorized into two groups: (1) the normal inner ear group, showing no abnormal signals in the inner ear, and (2) the auditory nerve strengthening group, wherein the enhanced scan revealed abnormal enhancement of the nerves, specifically the cochlear and vestibular nerves (Figure 1).

### 2.3 Audiological examination

All patients underwent pure tone threshold audiometry, acoustic immittance, distortion product otoacoustic emission, auditory brainstem response, and additional examinations before treatment to rule out related lesions and ascertain their level of hearing. The treatment effect was evaluated according to the results of pure-tone threshold audiometry performed 2 months after the disease onset.

### 2.4 Vestibular function examination

All patients underwent vestibular function examinations after admission, including the caloric test, vestibular evoked myogenic potential (VEMP), video head impulse test (vHIT), and vestibular autorotation test.

### 2.5 Degree and type of hearing loss

The degree of hearing loss was graded according to the pure-tone average at the following frequencies: (1) normal: ≤25 dB HL; (2) mild hearing loss: 26–40 dB HL; (3) moderate hearing loss: 41–60 dB HL; (4) severe hearing loss: 61–80 dB HL; and (5) profound hearing loss: ≥81 dB HL.

The types of hearing loss were categorized based on the frequency and degree of hearing loss as follows: (1) low-frequency type: hearing loss below 1,000 Hz (including at least 250 and 500 Hz), hearing loss ≥20 dB HL; (2) high-frequency type: hearing loss above 2000 Hz (including at least 4,000 and 8,000 Hz) hearing loss ≥20 dB HL; (3) flat type: all frequencies of hearing decline, 250–8,000 Hz (250, 500, 1,000, 2000, 3,000, 4,000, and 8,000 Hz), mean threshold ≤80 dB HL; and (4) total deafness: hearing loss at all listed frequencies with a mean threshold ≥81 dB HL.

### 2.6 Treatment method and duration

Treatment methods were applied in alignment with the treatment protocol outlined in the Chinese Guidelines for the (9), as follows: (1) low-frequency decline type: improved blood circulation using drugs (ginkgo biloba extract), glucocorticoids (methylprednisolone sodium succinate), and fibrinogen-reducing drugs (batroxobin); (2) high-frequency descending type: improved microcirculation using drugs (ginkgo biloba extract), glucocorticoids (methylprednisolone sodium succinate), and nutritive neuromedicine (mecobalamin); and (3) total frequency hearing loss (including flat decline and total deafness): drugs to improve inner ear microcirculation (ginkgo biloba extract), glucocorticoids (methylprednisolone sodium succinate), and fibrinogen-reducing drugs (batroxobin).

Treatment duration was defined as follows: intravenous drug therapy was ceased upon hearing recovery or if the patient failed the pure tone hearing test 10 days post-treatment initiation, and out-of-hospital oral medications (ginkgo biloba preparation, methylcobalamin) were discontinued. Oral drugs were discontinued if hearing did not improve during the two-week follow-up period.

### 2.7 Recovery

Recovery or therapeutic effects were classified as follows (9): (1) complete recovery: impaired frequency hearing threshold returned to normal, or up to the level of the other ear, or up to the level before the onset of the disease; (2) marked recovery: average hearing increased by more than 30 dB; (3) slight recovery: the average hearing improved by 15–30 dB; and (4) no change: mean hearing improvement <15 dB. For the analysis of the overall effect, instances of recovery, marked recovery, and slight recovery were collectively categorized as recovery.

### 2.8 Statistical analysis

Statistical analyses were performed using IBM SPSS Statistics for Windows (version 23.0; IBM Corporation, Armonk, NY, United States). Pearson’s chi-squared test was used to assess the curative effect, and non-parametric tests were used when the disease course and age did not follow a normal distribution. Logistic regression was used for the correlation analysis, and statistical significance was set at *p* < 0.05.

### 2.9 Ethics statement

The study design was approved by committee ethics board of the hospital (No. 2023–006- 6301).

## 3 Results

### 3.1 Clinical features

A total of 112 patients with ISSNHL, for whom complete data were available, were included. The male-to-female ratio was 1.15:1, and the left-to-right-sided ratio was 1.24:1. The mean age of the patients was 45 ± 12.73 years. Forty cases were accompanied by dizziness/vertigo; 21 cases were accompanied by underlying diseases, including hypertension; 16 cases had diabetes; seven cases had coronary heart disease; and some patients had more than one underlying disease. The degree of hearing loss was as follows: mild hearing loss in 17 patients (15.2%), moderate hearing loss in 32 patients (28.6%), severe hearing loss in 34 patients (30.4%), profound hearing loss in 29 patients (25.9%), low-frequency hearing loss in 15 patients (13.4%), high-frequency hearing loss in seven patients (6.3%), flat-type hearing loss in 62 patients (55.3%), and total deafness in 28 patients (25.0%). Among the 112 patients, MRI showed auditory nerve enhancement in 18 patients (16.1%). A comparison between the normal inner ear group and the auditory nerve enhancement group showed that the degree of hearing loss and type of hearing loss were significantly different (*p* < 0.05). The clinical characteristics of the two groups are shown in Table 1.

**Table 1:** Clinical characteristics of the patients.

Clinical feature	Abnormal (*n* = 18)	Normal (*n* = 94)	*p-*value
Male/Female	0.64:1 (7/11)	1.29:1 (53/41)	0.173
Left: Right	1:1 (9/9)	1.29:1 (53/41)	0.618
Age (years)	37 ± 24.04	46.5 ± 14.85	0.103
The course of disease (days)	10.38 ± 8.54	12.88 ± 12.77	0.751
Dizziness/vertigo	10 (55.5%)	30 (31.9%)	0.055
Caloric test	13 (72.2%)	49 (52.1%)	0.116
vHIT	7 (38.8%)	27 (28.7%)	0.390
VEMP	15 (83.3%)	64 (68.1%)	0.200
VAT	9 (50%)	54 (57.4%)	0.560
Degree of hearing loss**			< 0.001
Mild	0 (0%)	17 (18.1%)	
Moderate	2 (11.1%)	30 (31.9%)	
Severe	4 (22.2%)	30 (31.9%)	
Profound	12 (66.7%)	17 (18.1%)	
Type of hearing loss**			< 0.001
Low frequency	0 (0%)	15 (16.0%)	
High frequency	1 (5.6%)	6 (6.4%)	
Flat type	5 (27.8%)	57 (60.6%)	
Total deafness	12 (66.7%)	16 (17%)	

**p < 0.001.

vHIT, video head impulse test; VEMP, vestibular evoked myogenic potential; VAT, vestibular autorotation test.

### 3.2 Vestibular function

Of the 112 patients, 62 (55.4%) presented abnormal caloric test results, 79 (70.5%) abnormal VEMP results, 34 (30.4%) abnormal vHIT results, and 63 (56.3%) abnormal vestibular autorotation test results. Compared to the normal inner ear group, the auditory nerve enhancement group exhibited higher rates of abnormal results for the double-temperature test, VEMP, and vHIT, although the differences were not statistically significant (Table 1).

### 3.3 Auditory efficacy

Of the 112 patients, hearing returned to normal levels in 29 (25.9%); treatment was significantly effective in 19 (17.0%), effective in 13 (11.6%), and ineffective in 51 (45.5%) patients. Univariate and multivariate statistical analyses were conducted to ascertain the correlation between hearing efficacy and clinical features, such as sex, side of deafness, age, disease course, presence of dizziness/vertigo, underlying diseases, vestibular function, degree, and type of hearing loss (Tables 2, 3). Univariate analysis revealed correlations between efficacy and disease duration (*p* < 0.001), type of hearing loss (*p* < 0.05), and vHIT (*p* < 0.05). Multivariable analysis revealed that the curative effect was solely correlated with the duration of the disease at the time of treatment (*p* < 0.05). The total treatment effectiveness rate showed no statistically significant difference between the normal inner ear and the auditory nerve enhancement groups. However, the cure rate was significantly lower in the auditory nerve enhancement group (28.7 and 11.1% in the normal inner ear and auditory nerve enhancement groups, respectively).

**Table 2:** Single-factor analysis of hearing effects.

Clinical features	*χ*^2^/*Z*/*t* value	*p*-value
Sex	0.015	0.903
Side	0.008	0.929
Dizziness/vertigo	1.217	0.270
Age	−0.548	0.585
Course of disease**	−4.916	< 0.001
Underlying disease***	−0.271	0.786
Degree of hearing loss	−1.380	0.168
Type of hearing loss*	10.989	0.010
Inner ear MRI	0.010	0.919
Caloric test	1.116	0.291
VEMP	0.183	0.669
vHIT*	5.185	0.023
VAT	0.069	0.793

*p < 0.05; **p < 0.001; ***The number of underlying diseases in each patient.

MRI, magnetic resonance imaging; vHIT, vestibular evoked myogenic potential; VEMP, video head impulse test; VAT, vestibular autorotation test.

**Table 3:** Logistic regression analysis of treatment efficacy.

Clinical features	*p-*value	OR value	95% CI
Sex	0.592	1.418	0.396–5.084
Side	0.931	0.952	0.317–2.865
Age	0.249	2.899	0.475–17.702
Dizziness/vertigo	0.686	1.316	0.348–4.971
Course of disease (<1 week)**	0.001	-	-
1–2 weeks**	0.001	0.068	0.013–0.354
2–4 weeks	0.122	0.209	0.029–1.519
>4 weeks	0.965	0.956	0.131–6.957
Underlying disease***	0.808	1.311	0.148–11.574
Degree of hearing loss			
Mild	0.491	-	-
Moderate	1.000	-	-
Severe	1.000	-	-
Profound	1.000	-	-
Type of hearing loss			
Low frequency	0.145	-	-
High frequency	1.000	-	-
Flat type	1.000	-	-
Total deafness	0.999	-	-
Inner ear MRI	0.469	1.819	0.360–9.206
Caloric test	0.414	1.591	0.522–4.850
VEMP	0.746	0.824	0.256–2.650
vHIT	0.131	2.744	0.740–10.172
VAT	0.272	0.538	0.177–1.629

**p < 0.001; ***The number of underlying diseases in each patient; -As the number of people in the group is small, the OR value cannot be provided.

CI, confidence interval; OR, odds ratio; MRI, magnetic resonance imaging; vHIT, vestibular evoked myogenic potential; VEMP, video head impulse test; VAT, vestibular autorotation test.

## 4 Discussion

The auditory nerve, also known as the vestibulocochlear nerve, extends from the brainstem between the medulla oblongata and pons, enters the internal auditory tract along with the facial nerve, and divides into the anterior and posterior branches. The anterior branch is the cochlear nerve, and the posterior branch is the vestibular nerve. The vestibular nerve is further divided into the superior and inferior vestibular nerves. Congenital dysplasia of the auditory nerve, or acquired pathogenic conditions, can manifest as sensorineural deafness.

The pathogenesis of ISSNHL remains unclear; therefore, there is a lack of specific treatments. With the development of various technologies, modern MRI has been used to detect abnormal changes in the inner ear and nerves. Numerous studies have demonstrated that 3D-FLAIR sequences can effectively detect pathological changes in the internal auditory nerve. Previous studies have reported that Hunter’s syndrome is caused by the reactivation of the latent varicella-zoster virus in the geniculate ganglion that then infects the facial nerve (10). Since the facial nerve is adjacent to the vestibulocochlear nerve, the inflammatory response can extend to it. MRI of the facial and vestibulocochlear nerves in approximately 70% of patients with Hunter’s syndrome and local neuritis showed enhancement and no evidence of tumors, further supporting the idea that inflammation is the cause of hearing loss. In addition, activation of the varicella-zoster virus may cause simple cochlear neuritis, leading to ISSNHL (11, 12). Therefore, 3D-FLAIR sequences can be used to observe the auditory nerve and provide insights into its underlying pathology (2).

We retrospectively analyzed the clinical features and prognoses of patients with ISSNHL with abnormal enhancement signals in the auditory nerve on 3D-FLAIR MRI. MRI of 112 patients with ISSNHL showed that 16.1% of the patients had auditory nerve enhancement; however, they did not differ from the normal inner ear group in terms of sex, deafness, hearing loss profile, and age. The hearing loss in patients with auditory nerve enhancement was extremely severe (66.7%), which was significantly different from that in patients with a normal inner ear (*p* < 0.05). The cochlear function may be severely impaired in patients with auditory nerve enhancement.

No statistically significant differences were noted between the two groups regarding the incidence of vestibular symptoms and rates of various vestibular functional abnormalities. However, the incidence of vestibular symptoms and rates of abnormal caloric test, VEMP, and vHIT results were higher in patients with auditory nerve enhancement than in those with normal inner ears. The duration of vestibular impairment and central compensation also affected the results of vestibular function; gains can improve over time, depending on the central compensation rather than peripheral recovery (13). A longer duration of disease at treatment may yield normal vHIT results, and no statistically significant difference was observed between the normal inner ear and the auditory nerve enhancement groups.

Furthermore, no statistically significant difference was observed in the total treatment effectiveness rate between the two groups; however, the cure rate of the auditory nerve enhancement group (11.1%) was significantly lower than that of the normal inner ear group (28.7%). The effect of treatment on sudden hearing loss is subject to several influencing factors. Hence, we performed univariate and multivariable logistic regression analyses of the treatment effect and clinical factors. In the univariate analysis, vHIT, disease duration, and hearing loss type were the predictors of treatment efficacy. In the multivariate analysis, only the disease duration (especially within 2 weeks of treatment) was significantly negatively correlated with the treatment effect. No significant association was observed between dizziness/vertigo and poor hearing outcomes, which is consistent with previous findings (14–16). Therefore, in addition to the causes, the course of ISSNHL is important. Sudden hearing loss is a medical emergency; a detailed examination to determine its possible cause and early treatment are particularly important. The degree of initial hearing loss and disease duration exceeding 14 days were identified as independent risk factors for poor treatment efficacy (14, 17, 18). An association was noted between longer disease duration and lower treatment efficacy.However,our study had some limitations.The sample size was small,which probably limited its statistical power.Hence,future studies with a larger sample size are needed to verify our results.

In conclusion, in our study, 3D-FLAIR MRI of the inner ear revealed that auditory nerve enhancement was associated with more severe hearing loss, especially total deafness, in patients with ISSNHL. The cure rate of patients with auditory nerve enhancement was lower than that of patients with normal inner ears, although the overall treatment effectiveness rate did not differ. Additionally, the findings indicate that prompt treatment of ISSNHL, ideally within 2 weeks of disease onset, can aid hearing recovery.

## Data availability statement

The original contributions presented in the study are included in the article/supplementary material, further inquiries can be directed to the corresponding author/s.

## Ethics statement

The studies involving humans were approved by the Department of Otology Medicine, Shandong Provincial ENT Hospital, Shandong University. The studies were conducted in accordance with the local legislation and institutional requirements. The participants provided their written informed consent to participate in this study. Written informed consent was obtained from the individual(s) for the publication of any potentially identifiable images or data included in this article.

## Author contributions

XS: Writing – review & editing, Writing – original draft. LC: Writing – review & editing, Data curation. NH: Writing – review & editing, Data curation. WX: Writing – review & editing, Data curation. YW: Writing – review & editing, Data curation. KL: Writing – review & editing, Data curation. FD: Writing – review & editing, Data curation. HW: Writing – review & editing, Funding acquisition, Conceptualization. MW: Writing – review & editing, Writing – original draft, Supervision, Conceptualization.
